# Medical and Physical Intelligence

**Published:** 1802-11-01

**Authors:** 


### MEDICAL AND PHYSICAL INTELLIGENCE.

### [FOREIGN AND DOMESTIC.]

The earths, when combined with each other, frequently produce compoſitions which may be taken for new ſimple earths, and thus account for the errors that are ſometimes committed in the analyſis of ſtones and foſſils. Cit. Guyton had already made us acquainted with the action of earths on each other; Cit. Darracq repeating his experiments, confutes them, and adds ſeveral new ones, while he at the ſame time doubts the accuracy of others. The reſults of his experiments are as follow.

1. Cit. Guyton had thought, that on mixing together lime-water and the water of barytes, a precipitation was produced; which fact, however, never ſucceeded in Cit. Darracq's experiments, and he therefore believes, that the lime employed by that chemiſt contained a little ſulphuric acid, which occaſioned this precipitation.

2. The water of ſtrontian, of barytes, and of lime, mixed together, did not yield any precipitation.

3. The

3. The aluminous potash mixed with filiceous potash have produced a precipitation composed of filiceous earth and alum.

4. The filiceous potash is likewife precipitated with ftrontian and lime, when liquors containing thefe bodies in folution were mixed together.

It appears from thefe obfervations, that the alkaline earths form no combination with each other; whereas fuch a combination takes place between the non-alkaline earths, and between thefe and the alkaline, with exception of alum; or we may fay, that it is only the filiceous earth which poffeffes the property of detaching the earths from their watery diffolvents, and of forming with them terreous combinations.

5. Cit. Guyton had afferted, that the muriats of lime and of alum, when mixed together, yielded a precipitation not foluble in acids. Cit. Darracq, however, could not obtain this precipitation, and therefore, fuppofes, that this miftake of Guyton might have arifen from a fmall quantity of fulphuric acid, that always adheres to the aluminous earth, which is feparated from the alumen.

6. Cit Darracq could not obtain a precipitation from a mixture of muriat of lime with the muriat of barytes, and he alfo attributes that precipitation which Guyton has obferved, to the prefence of fulphuric acid.

7. It was impoffible to obtain any precipitation by feparately mixing muriat of magnefia with the muriat of alum and barytes, either by a mixture of the muriat of alum or of barytes, notwithftanding every poffible precaution, as propofed by Cit. Guyton; at leaft, not one earth would unite with another when earths were employed, diffolved in the fame acid.

---

Although it has been long known that the bulbs of feveral plants contain a great quantity of mucilaginous fubftance, no chemift has as yet endeavoured to feparate it, and to afcertain its nature by a chemical analyfis. Citizen *Le Roux*, of Verfailles, therefore, has opened the field of thefe inquiries, by analyfing the bulbs of Hyacinthus non fcriptus L. the vifcofity of which is very remarkable. On pounding a certain quantity of its roots in a ftone mortar, the pulpous mafs was carefully wafhed, and the water afterwards filtrated. On having evaporated this liquor to the confiftency of a fyrup, the refiduum became dry in a fhort time, amongft which a tranfparent fubftance was found in confiderable quantity, which had all the characteriftics of a real gum. A farther account of thefe experiments has been promifed by Mr. Le Roux.

---

Since Dr. Jenner's announcement of his difcovery of the value and importance of the Vaccine Inoculation, feveral refearches concerning the earlier hiftory of the difeafe have been made, by which it is found, that at different times before the prefent period, the moft obvious hints have been fuggefted, which might have led an impartial impartial obferver to expect the fame extenfive advantages from the inoculation of this difeafe as are now fo well underftood. What fatal coincidence of circumftances have we to accufe, by which the attention of medical men was formerly withdrawn from fo great and remarkable an object! The earlieft notice of the cow-pox, and of its power in preventing the infection of fmall-pox, has lately been found by Mr. Steinbeck to exift in a weekly publication, which formerly was publifhed at Gottingen, under the title of *Allgemeine Unterhaltungen*, i. e. General Entertainment: Gottingen, printed by Rofenbufch. In No. XXXIX of the volume for the year 1769 of this work, from page 305 to 312, is a differtation, by an anonymous author, who figns himfelf "an experienced husbandman," *On the Distemper amongst the Horned Cattle, and on some Passages in Livy*," of which the third fection contains the following interefting paffage: The author having defcribed what Livy calls *peftis*, queftions whether that which Livy comprehends under the general name of peftis, had always been the real plague, or fome acute fever, attended with eruptions, pox, purples, &c. "Though I do not venture, he proceeds, to give a decifive opinion on this fubject, it excited my attention, that fuch a plague as Livy defcribes, has, according to his ftatements, been common both to men and animals; a feature of the plague now not obferved. I have however, intimated before, that the *pestis* of Livy was probably nothing more than an eruptive fever, as it is faid to have been common both to men and animals, and as he exprefsly calls it, *scabies*. The cow-pox is not uncommon in the neighbourhood of Gottingen. It is true, that neither men nor animals die of this difeafe, though it is faid to be pretty fevere in fome perfons, which may be afcribed to our climate being colder, and thus rendering the poifon lefs venomous and dangerous. On this occafion I fhall mention, that people in the country near Gottingen, who have had the cow-pox, flatter themfelves that they are by it quite fecured from the infection of our common fmall-pox, as I have, upon accurate inquiries, frequently heard from creditable perfons." How happened it that this remark efcaped the attention of the phyficians of Gottingen, Hanover, and Germany? Gottingen and Germany would then have acquired the honour of this great Difcovery, which is now fo juftly obtained by England, and the lives of thoufands would have been faved, had any Phyfician, at that time, been poffeffed of the fagacity and perfeverance of a Jenner!

---

Mr. Giese, of Berlin, has made several experiments, in order to ascertain the presence and quantitive proportion of the benzoic acid in the urine of horses, which acid, according to the researches of Fourcroy and Vauquelin, is always to be found in the urine of herbivorous animals. The results of these experiments proved extremely different, as the benzoic acid could frequently not be traced traced at all, or in a great variety of proportion. It has likewise been intimated by the above French chemists, that the benzoic acid, found in the urine of herbivorous animals, especially in that of horses, might have originated from their food, and particularly from the vernal grass (*Anthoxanthum odoratum, L.*) which is frequently met with in hay. In order to enquire into the truth of this statement, Mr. Giese undertook a series of experiments with the above grass and with hay, with which it was mixed in considerable quantity. No traces of benzoic acid however could be discovered. He prepared an extract of it, which yielded a strong odour similar to that of melilot (*Trifolium melilot. officinal*), but after it had been digested with spirit of wine, and the mixture evaporated, not the least portion of benzoic acid could be traced in it. In oats this acid was likewise not to be found by a chemical analysis. Hence it appears, that the generation of the benzoic acid in the urine of horses and other herbivorous animals, seems to originate in the animal body itself; and it remains an interesting question for naturalists to determine in which state of the body it is particularly generated. Upon farther inquiry Mr. Giese found that the urine, in which he had discovered the greatest quantity of benzoic acid, had come from horses which had for a long time been sickly, whereas the horses whose urine contained no benzoic acid, or at least a very insignificant quantity of it, were apparently quite healthy. The benzoic acid, found in the urine of horses, is either in a free state, or neutralized with natron. Sometimes not a trace of benzoic acid is discovered in the urine of horses, which, however, yields a considerable quantity of calcareous earth. The urina cocta of horses seems in general to contain benzoic acid, which is rarely discovered in the urina cruda.

---

Cit. Tremery, a French Engineer of Mines, has lately publiſhed a Memoir, containing, what he calls, An *Examen of the electrical Phenomena which do not appear to accord with the Theory of the two Fluids.*

Among the facts which have been aſſumed as grounds to admit, with Franklin, the hypotheſis of a ſingle electric fluid, the moſt remarkable is the following: Having placed, between two metallic conductors, a card which touches each of them by one of its faces, in different points, he cauſed a ſtrong electrical diſcharge to paſs acroſs or over this apparatus; at the inſtant when it operated, a luminous track proceeded from the poſitive conductor, paſſed over the ſurface of the card, and penetrated it facing the negative conductor This took place even when the card was previouſly pierced, before the firſt of theſe two conductors.

It was inferred from this fact, that in order to admit the theory of the two fluids, we muſt ſuppoſe that a ſingle one of them may eſcape from the bodies that it contains, and produce light, whilſt the other remains inherent in its own. Cit. Tremery overthows this this reafoning by the following experiment: He placed the card and the two conductors under the recipient or receiver of a pneumatic machine; in proportion the denfity of the air contained under the recipient is diminifhed, the point where the card is penetrated approaches to the pofitive conductor; when the preffure was nearly half that of the atmofphere, the point of paffage was precifely in the middle of the two conductors. At each difcharge, a luminous track proceeded from each conductor, and fpread over each furface of the card, as far as the point of interfection.

Cit. Tremery concludes, from this experiment, that we muft confider the atmofpherical air, in its ordinary ftate, as refifting the paffage of the negative fluid, more than that of the pofitive fluid. Thefe refiftances diminifh, for each of thefe two fluids, with the denfity of the air, in different proportions, and much more rapidly for the firft than for the fecond.

From what precedes, Cit. Tremery deduces this general refult, that the ifolant faculty of idioelectrical bodies, fhould not be fuppofed the fame for both pofitive and negative electricities.

In proceeding according to this explication, it would be eafy to reconcile with the theory of the two fluids, the facts which his opponents fet againft him.

---

Cit. Guyton, Member of the National Inftitute and Director of the Polytecnic School, read at the meeting of the Inftitute, held 7th Fructidor, a Memoir, entitled Refearches on the Electric Pile of Volta, by Citizens Hachette and Deforues, Profeffors in the Polytecnic School.

This memoir contains two important facts, which tend to throw much light on the theory of electricity; the firft is, that an ifolated electric pile, or a friction machine of Nairn, pofitive and negative, and alfo ifolated, that is, only communicating with the atmofphere, is an inexhauftible fource of electricity. The fecond fact is, that many folid and dry fubftances, fuch as pure ftarch, or ftarch impregnated with different falts, may fupply the moift fubftances of the pile of Volta, and allow of a mode of conftructing piles, which, by the fimple action of fubftances placed above, become the conftant and inexhauftible fources of electricity.

---

The Inftitute of Health, of Gard, at their meeting at Nifmes, have offered, for the eleventh year, a gold medal of the value of 300 livres, for the beft Differtation on the following queftion: "Are there any phyfical or chemical means of deftroying the dangerous emanations which exhale from marfhes, or from ground newly drained, and of preferving from its influence thofe who are expofed to them."

---

The experiments and results of Mr. Lowitz, on the decoloration of vegetable liquors, and by pulverised charcoal, have been confirmed by some later experiments made by M. Duburga. The following circumstances may be relied on: Three ounces and a half of charcoal, purified by incandescence, mixed with 24 drops of of sulphuric acid, will purify $3\frac{1}{2}$ lb. of putrid water, without communicating any sensible acidity. The process consists in pouring the water upon this mixture, and afterward filtering it. It destroys the astringent principle; it absorbs fatty matters; it dissipates all fœtid smells, and may be used with advantage in cleansing musty casks; it has no effect on the smell of camphor, essences, ethereal oils, essence of orange, bark, &c.; it renders vinous liquors colourless; it diminishes scorbutic affections, sweetens bad breath, and whitens the teeth.

Cit. Vauquelin gives an account to the Societé Philomatique of a blue oxydated iron which was ſent from Salſburg to the Conſeil des Mines at Paris; it is of a light blue colour, and occurs in ſmall maſſes, that are concealed in the cavities of quartz and of a hard greeniſh ſtealites. It is friable and feels a little greaſy; it loſes its colour at the fire of the tube, and melts afterwards to a greeniſh glaſs. It is not to be diſcoloured either by acids or by weak alkalis, which circumſtance diſtinguiſhes it from lapis lazuli and the pruſſiat of iron. This blue ſubſtance, when digeſted with muriatic acid, communicates to it a ſaffron colour, whereby it is a little diſcoloured; but it loſes the colour entirely when it is diſſolved in this acid, in which it leaves behind a portion of ſiliceous earth.

On examining this ſolution, it will be found to contain alum, lime, and oxyd of iron; but neither manganeſe, nor ſulphurated hydrogen, nor phoſphoric acid can be traced in it, ſubſtances to which one might have attributed the blue colour of this oxyd of iron. It therefore remains to determine the cauſe of the remarkable colour of this oxyd; a colour, which we are not able at preſent to communicate to it by any known chemical proceſs. It is probable, that the iron is brought to a degree of oxygenation, that approaches the maximum.

Mr. Chausier employs a solution of oxygenated muriate of mercury, kept constantly in a state of saturation, for preserving animal substances from putrefaction. The preparations are to remain immersed in the solution several days, and then dried by exposure to light and air. After the process, they are no longer susceptible of being easily decomposed; they preserve their form, become possessed of a great degree of hardness, and are not subject to the attacks of insects.

List of Private Medical Classes to be opened at Edinburgh in the ensuing winter.

Anatomy and Surgery, Dr. Barclay.

Anatomy, Surgery, and Midwifery, Mr. Charles Bell.

Clinical Surgical Lectures in the Infirmary, Mess. Russell and J. Thompson.

Chemistry, Dr. Thomson.

Chemistry, Pharmacy, and Materia Medica, Mr. Murray.

Extemporaneous Prescription, Dr. J. Kirby.

Mr. Fox will commence his Lectures on the Anatomy and Diseases of the Teeth, in the Theatre of Guy's Hospital, on Tuesday, November 16, as half past five o'clock.

---

Dr. WALKER, Physician in Ordinary to the City of London Lying-in-Hospital, will publish, in a few weeks, General Observations on the Constitution of Women, and on some of the Diseases to which they are more especially liable.

---

On Wednefday, the 6th of October, Dr. MARCET, Phyfician to the City Difpenfary, was uanimoufly appointed Affiftant Phyfician to Guy's Hofpital, in the room of Dr. CURRY, who has lately been elected one of the Phyficians in Ordinary to that Inftitution.

---

Dr. JENNER has received a very valuable Diamond Ring from the Emprefs Dowager of Ruffia, together with a Letter, of which the following is a tranflation.

"SIR,

"THE practice of Vaccine Inoculation in England having been attended with the happieft fuccefs, which is well attefted, I have eagerly imitated that example, by introducing it into the charitable Eftablifhments under my direction.

"My endeavours having perfectly anfwered my expectations, I feel a pleafure in reporting my fuccefs, and in teftifying my acknowledgments to him, who has rendered this fignal fervice to humanity.

"This motive induces me to offer you, Sir, the Ring fent herewith, as a teftimony of the fentiments of efteem and regard with which I am,

"Yours, affectionately,

MARY."

*Paulowsky, Aug,* 10, 1802.

